# Renal Abscess Drainage Using a Novel Transgastric Endoscopic Approach: A Case Report

**DOI:** 10.7759/cureus.51294

**Published:** 2023-12-29

**Authors:** Abby Kunitsky, Kevin D Kunitsky, Chase Cavayero, Augustine Salami

**Affiliations:** 1 Department of Gastroenterology, Kansas City University, Kansas City, USA; 2 Department of Urology, Kansas City University, Kansas City, USA; 3 Department of Urology, Lee Health, Fort Myers, USA; 4 Department of Gastroenterology, Lee Health, Fort Myers, USA

**Keywords:** stent placement, renal abscess drainage, kidney, minimally invasive intervention, eus-directed transgastric endoscopy, interventional endoscopy, transgastric endoscopy, renal abscess

## Abstract

Renal and perinephric abscesses are rare purulent infections within or around renal parenchyma, typically treated with antibiotics or various procedural approaches depending on abscess size. In this case report, we describe the novel use of a transgastric endoscopic ultrasound (EUS)-guided technique with placement of a stent for drainage between a renal abscess and the stomach in a patient who had failed attempted percutaneous drainage twice and where an open surgical approach was deemed inappropriate. The patient presented with a chief complaint of left flank pain, with CT revealing a ~4 x 4 cm renal abscess in the upper pole of the left kidney. Urology, Infectious Disease, and Interventional Radiology were consulted. Following two failed attempts at percutaneous drain placement, the patient elected for EUS-guided transgastric stent placement for drainage. The stent was removed by postoperative day two after significant decompression of the abscess. He was advised to follow up outpatient with Urology to confirm full renal abscess resolution.

## Introduction

Renal and perinephric abscesses are uncommon infections with collections of purulent material within or around renal parenchyma. The incidence of these infections ranges from 1 to 10 cases per 10,000 hospital admissions [1-3]. Certain causes can include ascending dissemination of a urinary tract infection, hematogenous spread of infection from a distant site, or inoculation from regional lymphatics [1,2,4,5].

Diagnosing these abscesses poses a challenge as patients often exhibit a range of potential symptoms. The elusive nature of the diagnosis through physical examination necessitates the use of imaging techniques, with CT scans and ultrasound being the most frequently employed. Reliable diagnosis hinges on these imaging modalities due to the indistinct symptoms. The primary course of action involves the administration of antibiotics and percutaneous drainage, with the size of the abscess playing a pivotal role in determining the suitable therapeutic approach.

This case report describes a novel approach to renal abscess drainage using a transgastric approach which has been used before in Roux-en-Y gastric bypass gastric bypass patients, multiple pancreatic conditions, and gallbladder drainage [6-13]. To our knowledge, this is the first description of an endoscopic ultrasound (EUS)-guided technique with the placement of a stent specifically between a renal abscess and the stomach for drainage.

## Case presentation

**Figure 1:**
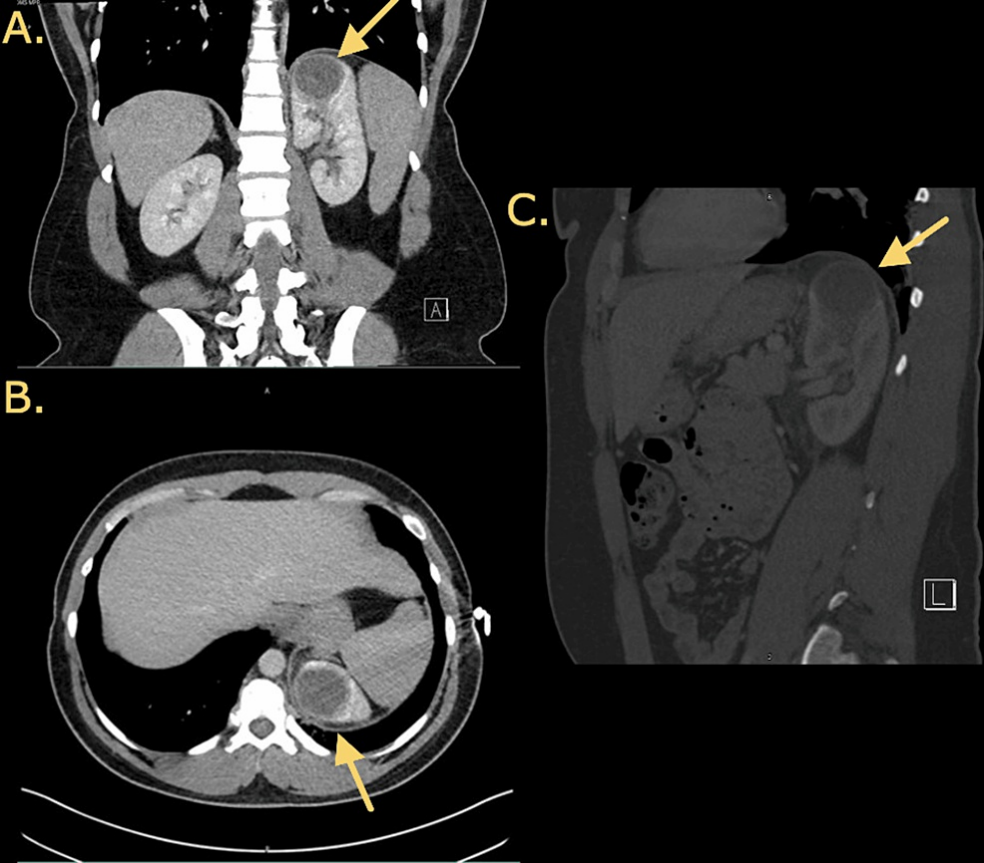
CT of the abdomen with contrast of the renal abscess showing a 4.1 x 4.1 x 3.8 cm rim-enhancing low-attenuation focus involving the upper pole of the left kidney. (A) Coronal view with the arrow indicating left renal abscess. (B) Axial view with the arrow indicating left renal abscess. (C) Sagittal view with the arrow indicating left renal abscess.

A 30-year-old transgender male with a past medical history of asthma and a kidney stone with no previous urologic evaluation presented to the emergency department via walk-in with a chief complaint of left flank pain for several days. He reported that he was seen one day before arrival at a different facility where he was diagnosed with a 4 cm left kidney mass versus an abscess seen on CTA of the chest. Labs at that time showed a white blood cell (WBC) count of 14.9 with creatinine of 1.1; he was administered ceftriaxone and ketorolac. The previous facility had recommended admission, however, the patient refused and was discharged with no further workup. The patient admitted to associated fevers and chills at home but denied dysuria, hematuria, abdominal pain, nausea, or vomiting. He also denied a history of intravenous drug use. Physical examination was notable only for mild left flank tenderness upon palpation but was otherwise unremarkable. The patient was tachycardic but was otherwise afebrile, and vitals were stable. Lab workup in the emergency department revealed a WBC of 17 with 12.9 absolute neutrophils, lactic acid of 0.6, and creatinine of 0.94. Urinalysis revealed pyuria with 30-40 WBCs, 3-5 red blood cells, small leukocyte esterase, negative nitrites, few bacteria, and trace protein. CT of the abdomen with and without contrast demonstrated a 4.1 x 4.1 x 3.8 cm rim-enhancing low-attenuation focus involving the upper pole of the left kidney consistent with renal abscess versus infected renal cyst (Figure 1). The patient was started on ceftriaxone. The patient’s left flank pain persisted despite supportive therapy. Urology, Infectious Disease, and Interventional Radiology (IR) were consulted for further management and possible drainage. 

Plans were made for drain placement due to the size of the abscess. Percutaneous drain placement was attempted by IR twice but was unsuccessful due to the location of the abscess in the upper pole of the left kidney near the diaphragm, pleura, and spleen. An open surgical approach was considered, but not felt to be indicated or appropriate. The case was discussed with a gastroenterologist with advanced endoscopy training who felt that the abscess could be drained using a unique EUS-guided technique with the placement of a stent between the abscess and stomach; he had previously used this approach to drain hepatic abscesses. Due to worsening pain, symptoms, and no improvement of leukocytosis despite antibiotics and supportive care, the patient elected this unique endoscopic approach. Two separately drawn blood cultures and one urine culture returned negative. The patient was started on IV piperacillin/tazobactam to cover for intra-abdominal infection as the intervention approach was through the stomach.

The patient was induced under general anesthesia, and the procedure was started by advancing a standard GIF-H1 90 gastroscope into the stomach. Erythematous gastropathy with gastric antral erosions and duodenal erosions were noted, with unremarkable findings of the esophagus and second portion of the duodenum. The curvilinear echoendoscope was then advanced into the stomach for a focused EUS examination. A 40 × 29 mm thick-walled unilocular anechoic structure on the superior left lateral aspect of the left kidney was identified, and the distance from this abscess to the gastric wall was measured to be 1.1 cm. The best window was felt to be at the lower end of the esophagus or the gastroesophageal junction. An avascular window was secured and the abscess was punctured using a 19-gauge Boston Scientific Slimline Expect needle. Overall, 5 ccs of purulent material was aspirated and sent for gram stain and anaerobic and aerobic culture. A 0.035-inch-long stiff shaft guidewire was then passed down the needle and looped into the abscess cavity. The needle was then removed and the wire was left in place. A 4 mm Hurricane biliary balloon was introduced over the wire and the transgastric access was dilated to about 2 mm. A 7 French by 3 cm double-pigtail plastic stent was then passed into the left renal abscess with the proximal pigtail in the gastric fundus just below the gastroesophageal junction, essentially creating a gastrorenal stent. The area around the stent was secured using the X-Tack helical tracking device running over 3-0 polypropylene sutures. A large amount of purulent material was noted to be extravasating from the stent into the stomach. The patient tolerated the procedure well. The plan was to leave the stent for at least one to two weeks, only removing it after imaging showed resolution of the abscess.

**Figure 2:**
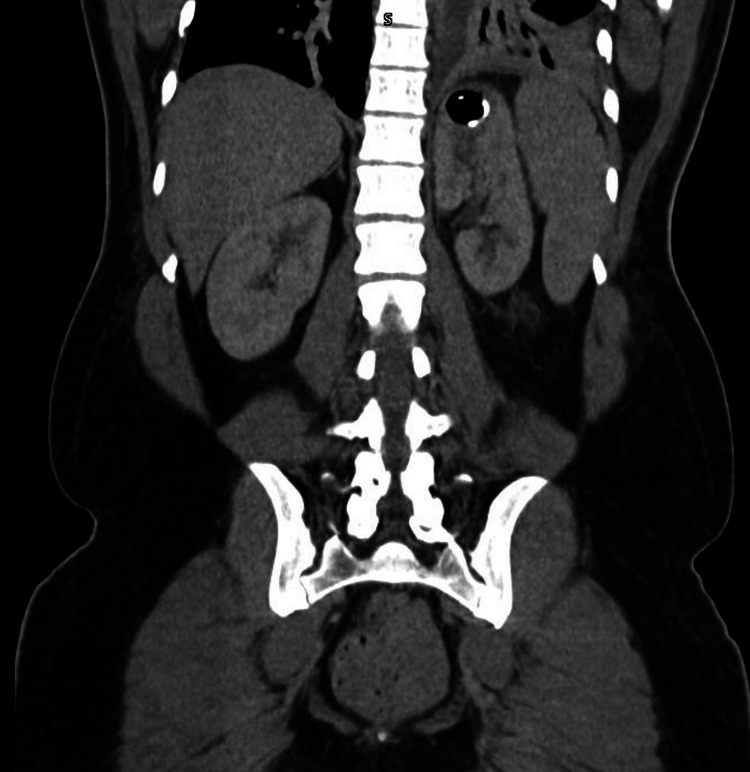
CT of the abdomen/pelvis with contrast, coronal view, showing decompression of the abscess with the stent in place.

**Figure 3:**
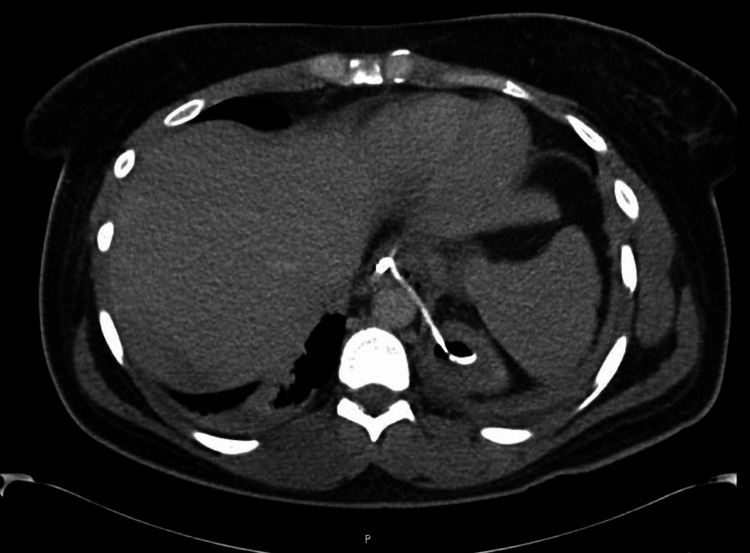
CT of the abdomen/pelvis with contrast, transverse view, showing the stent in place.

After the procedure, the patient was continued on IV piperacillin/tazobactam and started on vancomycin empirically. The patient refused the use of bilevel positive airway pressure (BiPAP) contrary to respiratory therapy recommendations. On postoperative day one, the patient developed severe left upper quadrant and left shoulder pain overnight. Vital signs remained stable, but leukocytosis worsened to 20. Due to concern about stent dislodgement, leak around the stent, or leakage of purulent material from the abscess into the retroperitoneum, a CT scan of the abdomen/pelvis was ordered (Figures 2, 3). Imaging demonstrated that the stent was correctly in place; however, it did show bilateral atelectasis, findings suggestive of mediastinitis and that the stent was transdiaphragmatic, believed to be causing the patient’s pain and leading to the worsening atelectasis. The patient underwent esophagogastroduodenoscopy that same day for re-evaluation which confirmed the correct position of the stent as well. Isovue was injected into the stomach and there was no extragastric contrast extravasation around the stent. The area around the stent was still buttressed with a single hemoclip. The patient was advised to use their incentive spirometer.

By postoperative day two, the abscess culture returned positive for scant *Escherichia coli*. Imaging showed significant decompression of the abscess, but the patient was still experiencing some discomfort, therefore, the stent was removed. This was done by grasping the stent with the snare loop on a standard gastroscope and removed intact through the scope. Two hemoclips were then used to close the previous stent tract at the gastroesophageal junction. Isovue was again injected to confirm no extravasation. The patient continued to experience some intermittent pain and hypoxia and was placed on BiPAP.

The next day, the patient was deemed to have bilateral pneumonia, atelectasis, and left-sided pleural effusion on further imaging. Leukocytosis improved, but the patient was still mildly hypoxic. Due to concern for infection, a chest Pleurx catheter was placed with significant drainage. The patient continued to have worsening pleural effusion the following day, so an esophagram was ordered to evaluate for a possible leak at the gastroesophageal junction/distal esophagus. The esophagram demonstrated no leak. His leukocytosis continued to downtrend, respirations improved, and drainage from the chest tube became minimal, hence, the chest tube was removed. The patient clinically improved significantly and was discharged home with ciprofloxacin and advised to follow up outpatient with urology for repeat CT to confirm renal abscess resolution. Pleural fluid cultures after patient discharge grew *Cutibacterium acnes*.

## Discussion

Renal abscesses generally occur more frequently than perinephric abscesses. Perinephric abscesses usually occur 60-90% of the time as a result of an intrarenal abscess extending into the perinephric fascia external to the renal capsule [1,2,5]. The incidence of these infections ranges from 1 to 10 cases per 10,000 hospital admissions [1-3]. Causes can include ascending dissemination of a urinary tract infection, hematogenous spread of infection from a distant site such as from *Staphylococcus aureus*, or inoculation from regional lymphatics [1,2,4,5]. One of the most common predisposing factors for renal abscesses is diabetes mellitus (associated in 33-62% of cases), followed by urinary tract infection, renal calculi, ureteral obstruction, urinary tract abnormalities, renal hematoma secondary to trauma, history of urological surgery, liver cirrhosis, chronic urinary retention, vesicoureteral reflux, or polycystic kidney disease [2,4,5,14-17]. Immunosuppressed individuals and intravenous drug users are also at an increased risk [2,5]. Reports show that these abscesses are primarily caused by gram-negative bacteria roughly 50% of the time, with the most common pathogens being *E. coli* and less commonly *Staphylococcus aureus*, *Klebsiella*, *Proteus*, and *Pseudomonas* [1,2,4,5,16,17]. Culture results are positive in blood in 12-16% of cases, positive in urine in 25-72%, and isolated pathogens in the pus in 50-61% [5,16-18]. In a 2016 study done at West China Hospital with 98 patients diagnosed with either renal or perinephric abscesses over 10 years, it was discovered that the average size of abscesses was 6.25 × 8.35 cm, with the largest abscess peaking at 17 × 20 cm [5].

Renal abscesses can be difficult to diagnose. Patients typically present with fever (50-100%), chills, flank/lumbar pain (53-76%), or abdominal pain; less likely symptoms can include dysuria (14-40%), nausea, vomiting, anorexia, lethargy, and weight loss [2,5,16,17]. The most common physical examination finding is costovertebral angle tenderness in 75-87% of cases [5,16]. Labs regularly show leukocytosis (usually less than 15), and abnormal urinalysis can present with pyuria, bacteriuria, or hematuria [2,5]. Azotemia can also be found in about 25% of cases [2]. Due to the generalizable symptoms and physical examination components of renal abscesses, imaging is paramount for a reliable diagnosis. The most useful imaging techniques for diagnosing a renal abscess include ultrasound and CT. Ultrasound can detect and localize the abscess with an accuracy of 70-93%, measure renal size, and determine if there is any obstruction of the collecting system [2,5]. CT helps define anatomical extent as well as fluid and gas involvement with an impressive accuracy of 92-96% [2,5,16]. Although MRI is technically more sensitive and specific than CT for renal lesions, due to its high cost and low accessibility, it is an unlikely first-line test for renal abscesses and is instead used to rule out renal malignancy [16]. Imaging frequently can mistake these renal abscesses as a benign cyst or central necrotic neoplasm; however, an abscess would have thicker, ill-defined walls, and sometimes even irregular contours [2]. Additionally, it is important to keep in mind the several other diagnoses that should be considered on the differential such as pyelonephritis and renal cell carcinoma [2].

The recommended initial treatment for renal abscesses includes adjunct antibiotics and percutaneous drainage. Antibiotic care is usually given parenterally for at least 10 days followed by oral antibiotics for an additional two to four weeks [2]. While antibiotics have been used as initial conservative treatment, different interventional and surgical drainage approaches have become an added requirement for better clinical outcomes than just antibiotics alone, with a 27 times higher cure rate with interventional treatment compared to conservative treatment [2,5,19]. Abscess size, location, and the type of abscess are some of the main determining factors for influencing the decision on antibiotic therapy alone versus additional interventional treatment, with larger sizes (>3 cm) and perinephric abscesses requiring more invasive therapies [15-17,20]. A retrospective review of 52 patients with renal abscesses revealed that antibiotic therapy alone resolved small abscesses measuring <3 cm 100% of the time; patients with medium abscesses measuring 3-5 cm treated with percutaneous drainage alone resolved 92% of the time; patients with large abscesses measuring >5 cm required more than one percutaneous drainage procedure 33% of the time or adjunct open surgical intervention 37% of the time [20]. It is also important to keep in mind that the sole use of antibiotic therapy, especially for perinephric abscesses, is not likely to be curative [2]. As an added benefit, interventional approaches can ultimately detect the cause, confirm the diagnosis, and obtain access for culturing the pus to guide antibiotic selection [5,17]. These interventions, such as percutaneous drainage, have contributed to lower case fatality rates, lower risk for intensive care unit admissions, and shorter duration of hospitalization [5,19]. Some uncommon but possible complications that can occur post-percutaneous drainage include pyopneumothorax, bacteremia, and fistula in the gastrointestinal tract [19]. Furthermore, in some cases, percutaneous drainage might ultimately require surgical exploration or nephrectomy in some patients if adequate drainage is not achieved [3,17,18]. Interestingly, a large significant predictor of prolonged hospital stays is if the patient has diabetes mellitus [17]. Overall, while past mortality rates used to be as high as 21-56%, now with more modern higher-quality imaging systems, advancement of antibiotics, and drainage techniques, the mortality rate has decreased to 0-14% [2,3,5,16,17].

This case report described a novel approach to renal abscess drainage using a transgastric approach. Transgastric approaches have been used previously for endoscopic retrograde cholangiopancreatography access in Roux-en-Y gastric bypass patients, pancreatic pseudocyst and duct drainage, pancreatic necrosectomy and gallbladder drainage with stent placement, among other procedures [6-13]. To our knowledge, this is the first description of an EUS-guided technique with placement of a stent specifically between a renal abscess and the stomach for drainage. This was performed by an advanced interventional endoscopist who had experience in EUS-directed transgastric endoscopy (EDGE) and had personally used this approach before to drain hepatic abscesses. Possible concerns for adverse events from this technique could include stent dislodgement, leakage around the stent, or leaking of purulent material into the retroperitoneum. These can be evaluated by CT imaging, direct visualization with esophagogastroduodenoscopy, or Isovue injection into the stomach to confirm no extravasation around the stent.

## Conclusions

Renal and perinephric abscesses can be difficult to treat using minimally invasive approaches based on the location of the fluid collection. In this case, our patient failed two attempts at drain placement by IR due to the location of the abscess near the diaphragm and pleura. The case was discussed with an advanced endoscopist who had experience using EDGE techniques and felt a transgastric approach could be used. Due to the invasiveness and lack of indications for an open approach, the patient elected for treatment using the novel transgastric approach with successful drainage of the abscess. With no other reports on this type of technique for the treatment of renal abscesses, it is difficult to conclude regarding the range of adverse events or other ways to minimize risks. Despite associated complications, the abscess was successfully drained with confirmation of resolution during outpatient follow-up. This type of approach can be used in certain difficult-to-treat patients but needs further refinement and exploration. Additional studies and attempts would be needed to come to a more definitive conclusion regarding the risks and benefits of this minimally invasive technique.
